# A novel inverter control strategy for maximum hosting capacity photovoltaic systems in distribution networks using power factor

**DOI:** 10.1371/journal.pone.0310301

**Published:** 2025-02-06

**Authors:** Ahmed Alfouly, Mohamed A. Ismeil, I. Hamdan

**Affiliations:** 1 Faculty of Engineering, Department of Electrical Engineering, South Valley University, Qena, Egypt; 2 Faculty of Engineering, Department of Electrical Engineering, King Khalid University, Abha, Saudi Arabia; 3 Department of Electrical Engineering, College of Engineering and Information Technology, Buraydah Private Colleges, Buraydah, Kingdom of Saudi Arabia; HJNU: Hanjiang Normal University, CHINA

## Abstract

The conventional inverter is undergoing a transformation into a smart inverter, driven by the expanding penetration of Photovoltaic (PV) power production in Low Voltage (LV) systems. The adoption of smart inverters is on the rise. Power companies are keen on integrating them into their networks to acquire essential frequency and voltage support as required. These inverters actively exchange actual and reactive power in connection with the grid, altering the system’s operational state. This dynamic behavior within the distribution level of power networks might give rise to unprecedented issues. This paper investigates the influence of diverse connection prerequisites that explore the methods for determining the Hosting Capacity (HC) of PV solar systems and their applicability within the low-voltage utility grid. Smart inverters provided with different Volt-VAr and Power Factor (PF) regulation capabilities are analyzed using MATLAB SIMULINK. The outcomes reveal a notable augmentation in the network’s HC. This progress improves the grid’s attributes, and the incorporation of smart inverter functionalities stands to considerably facilitate incorporating PV solar installations into electrical networks.

## Introduction

The increasing energy demand has led to thinking of alternative and non-exhaustible sources. On the other hand, this preserves the environment by reducing carbon emissions. By 2050, it is predicted that renewable energy sources will account for 86% of power production and 2/3 of the world’s energy consumption [1]. Renewable energy sources, for example photovoltaic (PV) systems, are gaining popularity as distributed generation resources. Solar power stations equipped with multiple photovoltaic panels have achieved success in connecting to different distribution networks, and they can also be connected to high-voltage networks through different power transformers. Furthermore, traditional fossil-fuel-powered turbines that are being retired are being in a place of similar power derived from renewable resources [2]. The growth of PV arrays in a low-voltage distribution network produces voltage rise issues at the connection feeder and low-voltage infractions of operational efficiency constraints and system reliability [3,4]. This violation has been acceptable limits, the acceptable limit means that the PV generation does not exceed the capability of hosting solar panels of a feeder.

The PV hosting capacity has generally defined a maximum connectable solar power output to the grid without impacting the system’s regular operation [5]. This definition depends on several factors, including voltage rises that cause the system’s power flow to reverse, thermal overloads of conductors and transformers, and unbalanced voltage. Solar PV system evaluation of an actual network due to the impact of several elements, similar to the topology, feeder characteristics, degree of network loading, and location of PV constitute installations [6]. The primary challenges that hinder the maximum connected capacity of PV arrays often involve voltage elevation in practical LV distribution systems, leading to the breach of legal voltage limits [7–9].

Smart inverters, formerly referred to as sophisticated inverters, have caused a fundamental change when it comes to the implementation of Distributed Energy Resources (DER) [9]. These inverters can carry out several tasks including both reactive (Volt-Var) and active power (Volt-Watt) regulation, moreover, voltage regulation, PF control, real power limits, ramp-rate regulation, fault ride-through, and frequency control are some of the processes involved in converting electricity from DC to AC. Various grid support services are currently being demonstrated using smart inverters on actual distribution and transmission systems in several nations [10].

The challenge of managing voltages and reactive energy fluxes throughout the entire distribution system prompted the creation of the Volt-Var control system. Volt-Var evaluates all voltage control and VAR regulation devices, such as voltage regulators, on-load tap changers, and selectable shunt capacitors, to minimize network losses and device operating expenses. This is achieved by considering operational constraints, including voltage and line flow limitations, in order to establish the most efficient set of control operations [11].

The goal of maintaining efficient and secure power distribution systems through voltage (Volt) and Volt-VAr regulation is the main issue of stability during hosting capacity. Most distribution systems use power circuits and have a topology resembling a tree with far-flung nodes. The substation magnitude often produces low voltage. The grid’s voltage batteries, regulators, and capacitors work better thanks to Volt-Var, which also maintains the voltage profile at every node and lowers power delivery losses (node). Volt-Var is therefore one of the essential elements of distribution automation [9].

One approach of voltage control in PV-heavy distributing systems has drawn a lot of attention: the Volt-VAr management of smart inverters. Voltage control may be quickly and continuously provided by smart inverters, in contrast to grid voltage regulators like on-demand tap switchers and selectable shunt capacitors [12]. Additionally, even when the feeder’s real and reactive energy fluctuates, a smart inverter employed to maintain the PF remains constant. Furthermore, researchers have investigated strategies for the purpose of expanding hosting capability grounded on smart inverters [13–16]. Time series, stochastic, and deterministic are three different hosting capacity approaches. These techniques look at how a high proportion of solar systems in these networks impacts the voltage amplitude and how much the transformers, wires, and lines are loaded. They are investigating hosting capability measurement methods for solar power in low voltage electricity power. However, other utility occurrences get reduced consideration. Deterministic, stochastic, and time series methods are used to assess and investigate the impacts resulting from rising solar PV penetration levels on voltage as well as loading [9,17]. Excessive DG penetration may cause several issues, which encompass overvoltage, excessive heat buildup, problems related to power quality, and safeguarding the system issues if it is not adequately assessed. Additionally, it is possible to view overvoltage as the primary issue with high DG penetration [18,19]. There are numerous alternatives for controlling, like Volt-Watt, Volt-VAr, and combined controls. The Volt-VAr regulation provides the best efficiency and is more affordable than the merged Volt-VAr and Volt-Watt control, according to a comparative examination of photovoltaic Solar hosting capacity increase employing these two control systems [3,20]. The term hosting capacity appeared recently and became a hot topic, especially with the expansion of the use pertaining to solar power and wind power. This prompted a lot of those interested in energy to increase the capacity used from renewable with new methods of control, as was found in the comparison between the Volt-VAr and STATCOM in HC systems, it is found that the outcomes of Host Capacity by using Volt-VAr regulation in low voltage distribution grid is better than STATCOM are presented in reference [18], and also when comparing between PF & Volt-VAr controls discovered that the PF control is more stable than Volt-VAr control, hence the PF control is better than STATCOM [21]. Considering a group of potential sites, the approach calculated both the highest generation capacity achievable by a solar generating station and the optimal Volt-VAr configurations. The outcomes unveiled the ideal feeder positioning for an individual solar energy system setup. Moreover, in contrast to the scenario where the PV inverter functions with a power factor of unity, the Volt-VAr regulation graph values ascertained through the method of enhancing performance elevated the highest achievable generation capacity by 45.21% [22]. Moreover, the efficiency of distribution networks while increasing the capacity for hosting photovoltaic (PV) energy has been increased. On the other side, the optimal functioning of capacitor banks has been considered on-load tap changers in substations, voltage controllers, and altering the network structure using both radial and closed-loop operational configurations. In addition, the regulation of voltage and reactive (Volt/VAr) power has been considered [23,24]. The hosting capacity increase can be controlled and evaluated by the smart inverter and the energy storage system. While this system offers the most effective approach to enhancing hosting capacity, its cost is higher due to the incorporation of batteries [25,26]. The author employs an analytical technique grounded in HC solution theory to establish the highest quantity of solar photovoltaic installations that distribution networks can support. Particularly, a numerical model for the HC issue is used for locating the global ideal that drives the theory of resolution [27]. The HC of photovoltaic systems for a practical distribution feeder in Ontario on two Solar panel farms has been enhanced and sophisticated inverter Volt-VAr management is in place [28]. Previous works do not mention the PF control of the HC for the PV system. However, a few of the works are interested in the reactive and actual power of the PV inverter. The reactive power from the PV inverter is more effective because it enhances the voltage bus at the PCC. Hence, the power factor is effective pertaining to the electrical utility. So, in this article, the PF control is used to improve the HC in the PV system array that is linked to a low-voltage distribution grid. Also, the hosting capacity has been achieved with different methods of control such as PI, Volt-Var control, and PF control in different cases without any fault occurring and with (single line-ground, two line-ground, three line-ground) faults. The system parameters like real energy, reactive energy, bus voltage, apparent power, and PF are investigated based on the studied control approaches. In addition, different control methods have been presented and analyzed to select the best control to boost the hosting capacity for the PV systems linked to the power grid at a low voltage distribution grid.

The remaining sections are organized as follows: Part II introduces the PV solar system analysis. Part III discusses the Volt–Var and Power Factor (PF) control. Part IV presents the results from simulations. Part V outlines future work. Finally, Part VI highlights the conclusions drawn from the findings.

## 2. PV solar system

The basic parts of the PV generating system are the inverter and PV arrays. To establish a solar cell that has the necessary voltage and current for practical utilization, the cells are linked in series, parallel, or both configurations.

The equations describing the behavior of PV panels have been presented in [29]. The output current *I*_*pv*_ of a PV module identified as:

$${I_{PV}=I}_{Ph}-I_{O}\left[\exp\left(\frac{V_{PV}+I_{PV}R_{S}}{a}\right)-1\right]-\frac{V_{pv}+I_{pv}R_{S}}{R_{Sh}}$$ (1)

where, *I*_*PV*_ Output Current (A), *I*_*O*_ the diode’s leakage current present (A), *V*_*pv*_ Voltage MPPT (V), *R*_*Sh*_ Shunt Resistance and *R*_*s*_ Series Resistance.

The key interface components connecting the PV system and the grid consist of a DC/DC converter and a DC/AC inverter. The MPPT algorithm is used to control the DC/DC converter in order to guarantee optimum generating power from the photovoltaic source. The produced electricity is conveyed to the AC aspect with the help of an inverter. Some examples of inverter control involve tasks such as grid synchronization, maintaining DC connection voltage balance, and regulating active/reactive power. It is common practice to modify the inverter current to manage both active and reactive power control [29].

**Fig 1:**
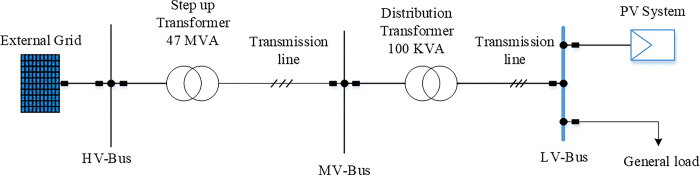
One-line schematic of the test system.

The proposed control will be validated based on the system shown in Fig 1. The setup comprises a simple low-voltage distribution network supplying a 100 kVA PV system and a 10 kVA aggregated load located 5 km away from a feeder’s end. The comprehensive specifications of the photovoltaic system, which is powered up to its maximum level are summarised in Table 1. It has the appropriate quantity of series and parallel PV arrays with PV capacity. The operation conditions for the PV system are selected to be standard conditions which are 1000W/m2 for irradiation level and 25Co for temperature.

**Table 1:** Parameters of the solar photovoltaic system [30].

Characteristics	Magnitude
Solar Photovoltaic Energy Rating	100kW
Photovoltaic module	305W
Rated Current of the model	5.58A
Rated Voltage of the mode	54.7V
Radiation Limitations	1000W/m^2^
Quantity of Sequential Models	5
Quantity of Parallel Models	66
Thermal Conditions	250C
Distribution Transformer	100Kva/0.4/25kV
Feeder Length	5km

## 3. Proposed power factor control strategy based on smart inverter

The smart inverter is distinguished from the traditional inverter by its ability to control many outputs of the PV system connected to the electrical network, such as real/ reactive power mitigation. In addition, the following features are popular within smart inverters designed for residential-scale applications:

Upper generation threshold.
Constant power factor.
Real/reactive power with voltage control.
Combines active and reactive power alongside voltage and frequency controls.

These controllers regulate voltage by lowering and supplying active power or compensating reactive voltage [25].

**Fig 2:**
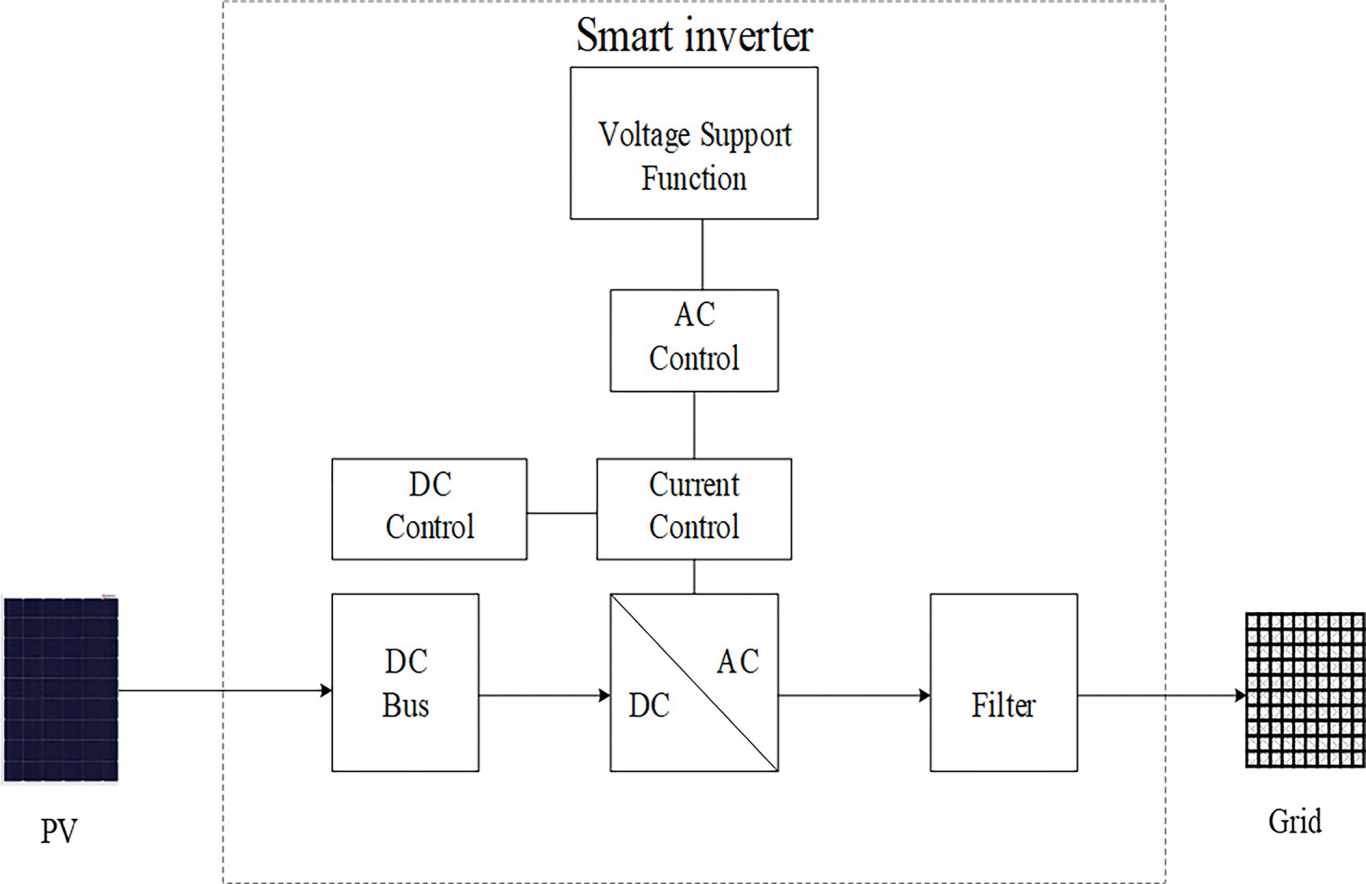
Block schematic of smart PV inverter.

Smart inverters allow for two-way dialogue involving utility control centers. Thanks to cutting-edge technologies like voltage and frequency detectors. Smart inverters can also detect power grid irregularities and communicate their findings to utility operators. Below, in Fig 2, is a general block schematic of a smart PV inverter system [31].

The smart inverter has many functions compared to traditional functions. Table 2 illustrates the comparison of the traditional inverter to smart inverters.

**Table 2:** Comparison of smart and traditional inverter [32].

Smart Functions	Traditional Inverter	Smart Inverter
Connect and play	×	√
Autonomy	×	√
Adaptability	×	√
Self-awareness	×	√
Cooperativeness	×	√
Connect/Disconnect	×	√
Reactive power output	×	√
Power Factor Dynamic	×	√
Dynamic regulation of battery charging	×	√
Identification of threats and dangers in advance	×	√
Time management	×	√

A set of guidelines has been established by the Electric Power Research Institute (EPRI smart inverter project) the smart inverter’s abilities for controlling local voltage, frequency, and network protection have been established. Table 3 depicts the description of smart inverter functions based on their intended usage [32].

Voltage restrictions that typically restrict capability for hosting in LV distribution systems can be mitigated by modifying either or both reactive and real power utilizing smart PV inverters’ Volt-VAr and Volt-Watt regulation [25]. Volt-VAr mode dynamically adjusts voltage at the location of control by delivering or injecting reactive power under Undervoltage and overvoltage situations, respectively. Voltage-VAr mode and Watt-VAr mode can be combined or modalities of reactive power prioritization (VAR prioritization). The photovoltaic (PV) inverter has the capability to produce reactive power when employing Volt-VAr regulation under VAR priority state. However, the production of actual power is restricted because of the need for reactive energy. Volt-VAr regulation reduces the portion of the inverter’s useful power if the margins for reactive power cannot deliver or consume the necessary voltage regulators (VARs) and converters with VAR priority [33]. voltage control can’t be possible with Volt-VAr mode if the inverter uses all active power. The behaviour of the inverter at over-voltage conditions reduces active power output in reaction to overvoltage conditions [34].

**Table 3:** Different features of smart inverter functions [25].

Voltage support features	characteristics that enable frequency	Features that aid with network protection (Response to disturbances)
Voltage-reactive power control	Watt-Frequency Control	Control over connectivity
Voltage-active control	Specifications for Ride-Through at Low/High-Frequency	Low/High-Frequency Conditions for Ride-Through
Real power-reactive power	Restricting the highest actual power	the specifications for both low- and high-voltage ride-through
Fixed PF Control	The functionality for controlling the rate of charging or discharging ramps	Behavior of temperature-PF
Actual power factor control		
Dynamic reactive energy factor adjustment		
Restricting peak actual power output		
Functionality for controlling charge/discharge ramp rates		

To standardize new functions in smart inverters, the IEEE Std. 1547–2018, AS/NZS 4777.2–2015, California Rule 21–2018, and Hawaii Rule 14–2018 (Revised Sheet No. 34B-21) have been used. as well as other standards, have recently been updated. Table 4 summarises these changes. An overarching trait of all norms is that voltage and reactive power management capabilities cannot coexist harmoniously; they can only be activated singularly, with one of these functionalities taking precedence at any given time [35].

**Table 4:** Regulation of voltage and reactive power should be improved, according to various standards [25].

Control modes	Standards			
	IEEE1547_2018	AS/NZS 4777.2_2015	California Rule 21_2018	Hawaii Rule 14_2018
Continuous PF	✓	✓	✓	✓
Reactive Voltage (Volt-VAr)	✓	✓	✓	✓
Voltage-real power (Volt-Watt)	✓	✓	✓	✓
Steady reactive power	✓			
Real, reactive power	✓			
Real power—Power factor		✓		
Frequency-Power	✓		✓	

Smart inverters can mitigate the consequences of growing PV adoption by applying actual power limiting and/or reactive compensation. These devices can be used to manage the inserted active power level (Volt-Watt control) or to provide an approach, varying according to the voltage level, to give a variable (Volt-VAr mode) [25].

The power factor holds significant prominence factors affecting energy production systems. The smaller the power factor becomes, the higher the reactive power, and this leads to low efficiency and greater fuel consumption in thermal plants [35]. Therefore several methods have emerged to improve the power factor such as capacitor banks, synchronous generators, static VAr compensators, and phase advances [36].

The equations of power factor are used in this paper [37]. Eq (2) is express the reference active power obtained by the product of the direct axis for reference voltage and the current from the PV system. Also, Eq (3) describes the reference reactive power that is absorbed or injected from the PV inverter to maintain the bus voltage and it is obtained by product quadrature axis reference reactive voltage and current. Moreover, Eq (4) presents the apparent reference power, which is the outcome of actual voltage and actual current. Where dividing the reference active power by apparent reference power led to obtaining the reference power factor as presented in Eq (5). Also, Eqs 6–9 describe the real, reactive, apparent power, and power factor measured at the point of connection PV solar system.

$$P_{ref}=V_{d-ref}*I_{d-ref}$$ (2)

$$Q_{ref}=V_{q-ref}*I_{q-ref}$$ (3)

$$S_{ref}=\sqrt{{\left(P_{ref}\right)}^{2}+{\left(Q_{ref}\right)}^{2}}$$ (4)

$${PF}_{ref}=\frac{P_{ref}}{S_{ref}}$$ (5)

$$P_{mes}=V_{d-mes}*I_{d-mes}$$ (6)

$$Q_{mes}=V_{q-mes}*I_{q-mes}$$ (7)

$$S_{mes}=\sqrt{{\left(P_{mes}\right)}^{2}+{\left(Q_{mes}\right)}^{2}}$$ (8)

$${PF}_{mes}=\frac{P_{mes}}{S_{mes}}$$ (9)

where, *P*_*ref*_, *Q*_*ref*_, *and S*_*ref*_ are the references real, reactive, and apparent power of the system, also *P*_*mes*_, *Q*_*mes*_, *and S*_*mes*_ are the measurements real, reactive, and apparent power of the system. $V_{d-ref},I_{d-ref},V_{q-ref}\;and,I_{q-ref}$ are references direct axis and quadrature axis voltage and current respectively. $V_{d-mes},I_{d-mes},V_{q-mes},and,I_{q-mes}$, are measurements direct axis and quadrature axis voltage and current respectively, *PF*_*ref*_, *PF*_*mes*_ are reference and measurement power factor respectively.

**Fig 3:**
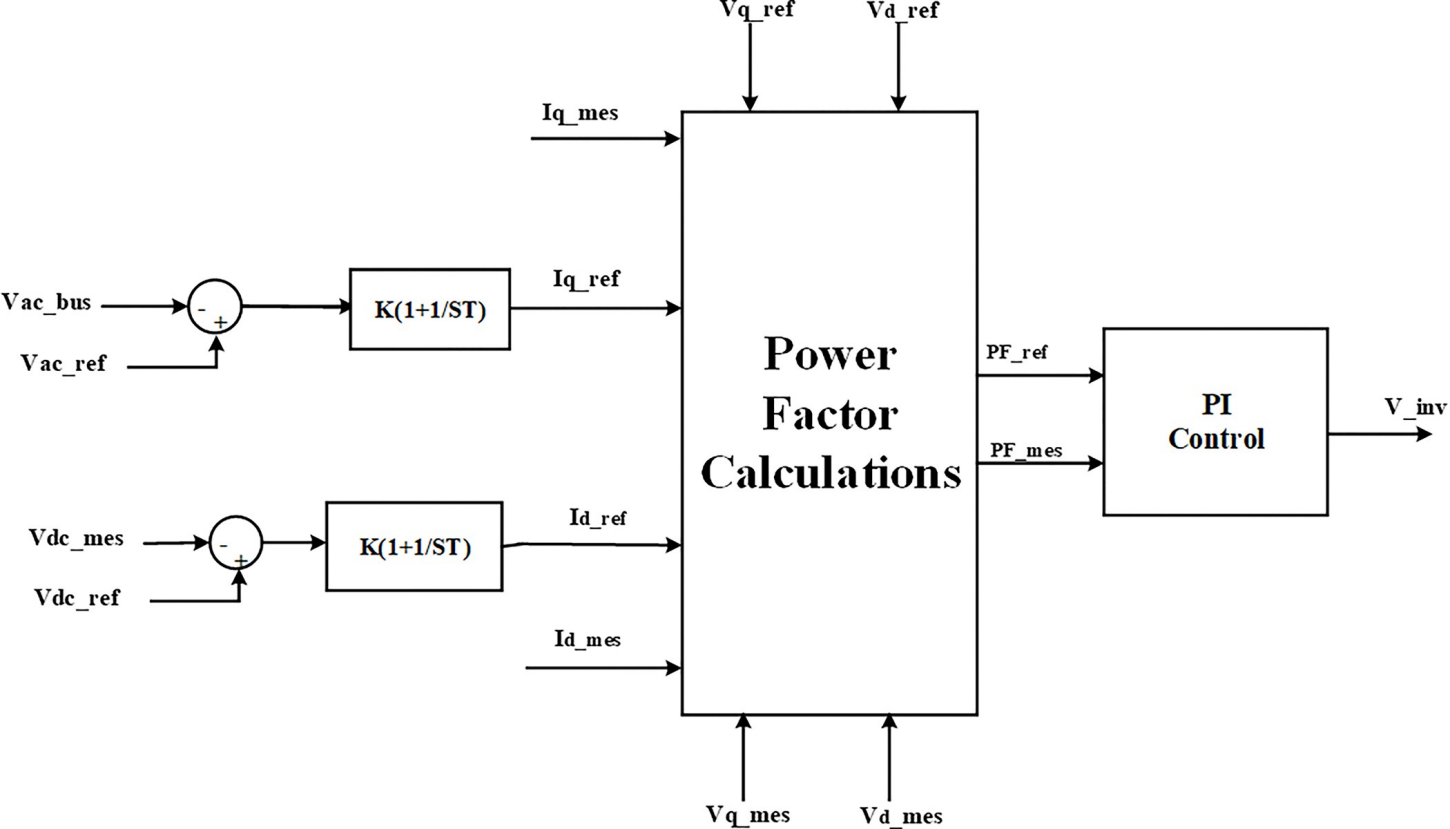
Schematic power factor controller in solar system.

By using Eqs (2) to (9), the controller for PV solar inverters is created with power factor control as shown in Fig 3.

## 4. Hosting capacity calculations

**Fig 4:**
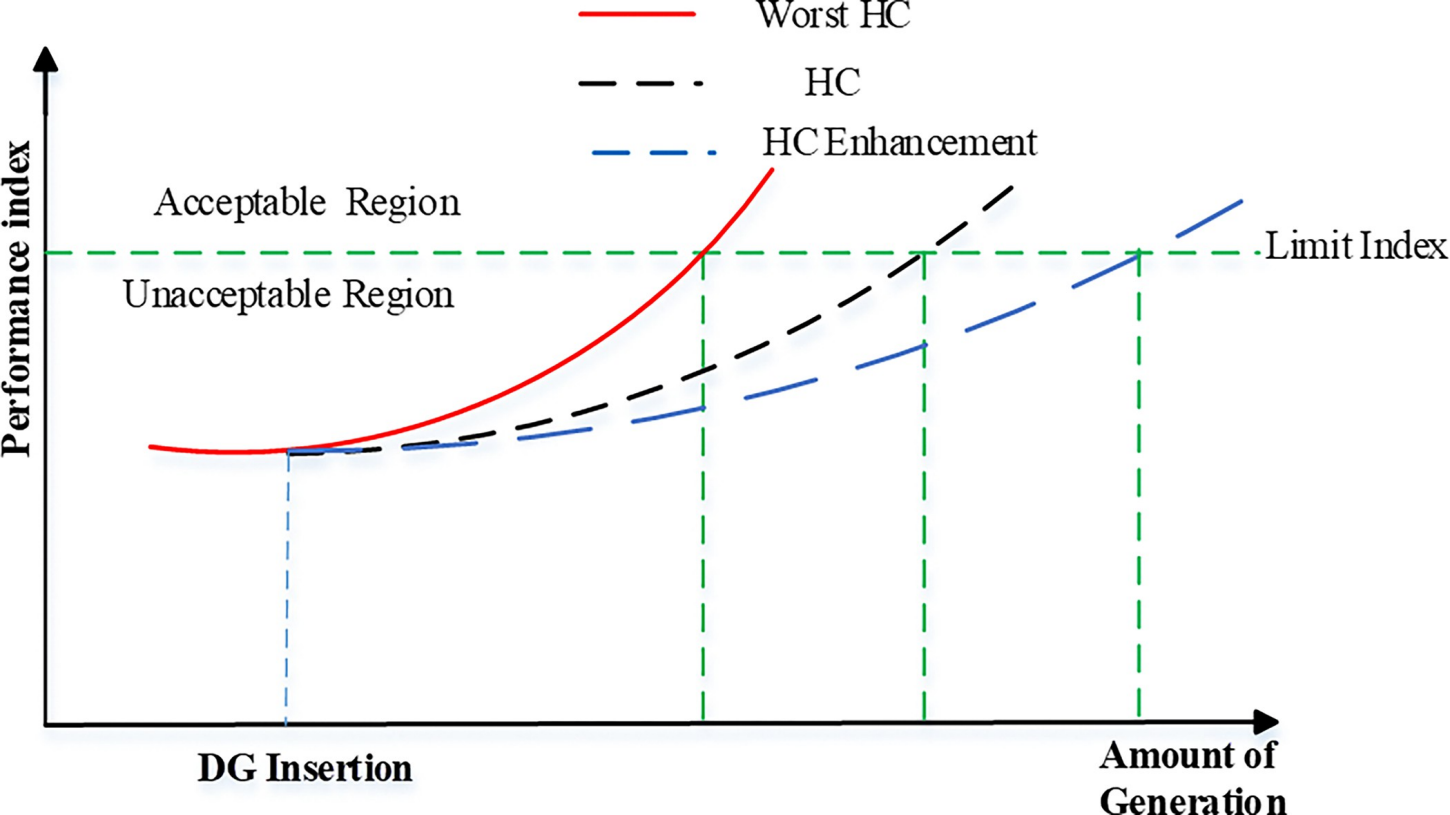
The idea of HC and the outcomes of its development.

The performance of the power system network changes due to adding loads or generation stations. The HC can be Characterized as the quantity of power that has the potential to be introduced into the Distribution network excluding compromising the quality or reliability of the system for consumers [34]. In other words, the network that has a good HC when the network can handle the amount of excess local generation and is typically constrained by distribution networks and the components that make them up. This restriction can go no higher than the hosting capacity. Exceeding this limit can lead to the overloading of components, which decreases their lifespan or, results in system failure. According to formal definitions, HC is the greatest distribution generation that a system can support while still operating under design guidelines and network design practices. Fig 4 depicts the HC concept and increasing the network’s HC could increase the amount of distributed generation additions while still adhering to the system’s performance constraints. Hence, the power can be hosted from RES without losing the functioning constraint of the electricity distribution system which means that the power penetration level of RESs is driven within a safe level.

Relevant performance indices, which will differ depending on the study, must first be identified to assess the hosting capacity. These metrics will show whether the network’s working circumstances are adequate. The highest value a performance index can attain is its HC limit. The electrical grid will be compelled to run in intolerable conditions once this limit is reached [38].

The following factors affect the hosting capacity: Overload limits, over-current limitations, transformer restrictions, frequency limits, protection limits, consumer and customer service parameters, and economic constraints based on investment costs are the overvoltage limits that have the greatest impact [39]. Conductor kinds, higher voltage threshold, degree of thermal loads, and the location of photovoltaic are all considered while conducting a capacity study.

The hosting capacity equation can be represented as:

$$HC\%=\frac{HC(with)-HC(without)}{HC(without)}$$ (10)

where, HC (with) represents the apparent power when the control is being used, and HC (without) represents the apparent power when the control is not being used [3]. Where the PV inverter gives actual power and injection the reactive power into the network at low voltage, hence the loads reduce absorb the reactive power from the network, also constant the voltage bus at the PCC, and reduce the cost generation of reactive from the network, and also when overvoltage occurs, the actual power from PV is fixed but take in reactive power from the utility to maintain the voltage at connection PV system is a constant magnitude, hence the hosting capacity should be equal the apparent power not an active power.

Achieving system stability involves mitigating voltage fluctuations and minimizing electricity losses within the utility, while also enhancing the capacity of a solar system. Although a battery system can provide superior hosting efficiency in this context, it comes with several drawbacks, such as the considerable costs and ongoing maintenance required for the battery.

The structure of the traditional power network was distinctive as the power flowed in just one direction from generation to transmission to distribution. There is a need for stable, continuous power electrical energy consumption is rising; nevertheless, reversing the power flow is the issue with hosting capacity.

Smart inverters can mitigate the consequences of growing PV adoption by incorporating active power limiting and/or reactive balancing. Depending on the voltage level, these devices can act on the real power constraint (Volt-Watt regulation) or reactive reparations to offer adjustable regulation (Voltage-Var control). The storage system can be configured to accept additional solar energy produced while assisting in maintaining the grid’s dependability [37].

## 5. Results and discussion

**Fig 5:**
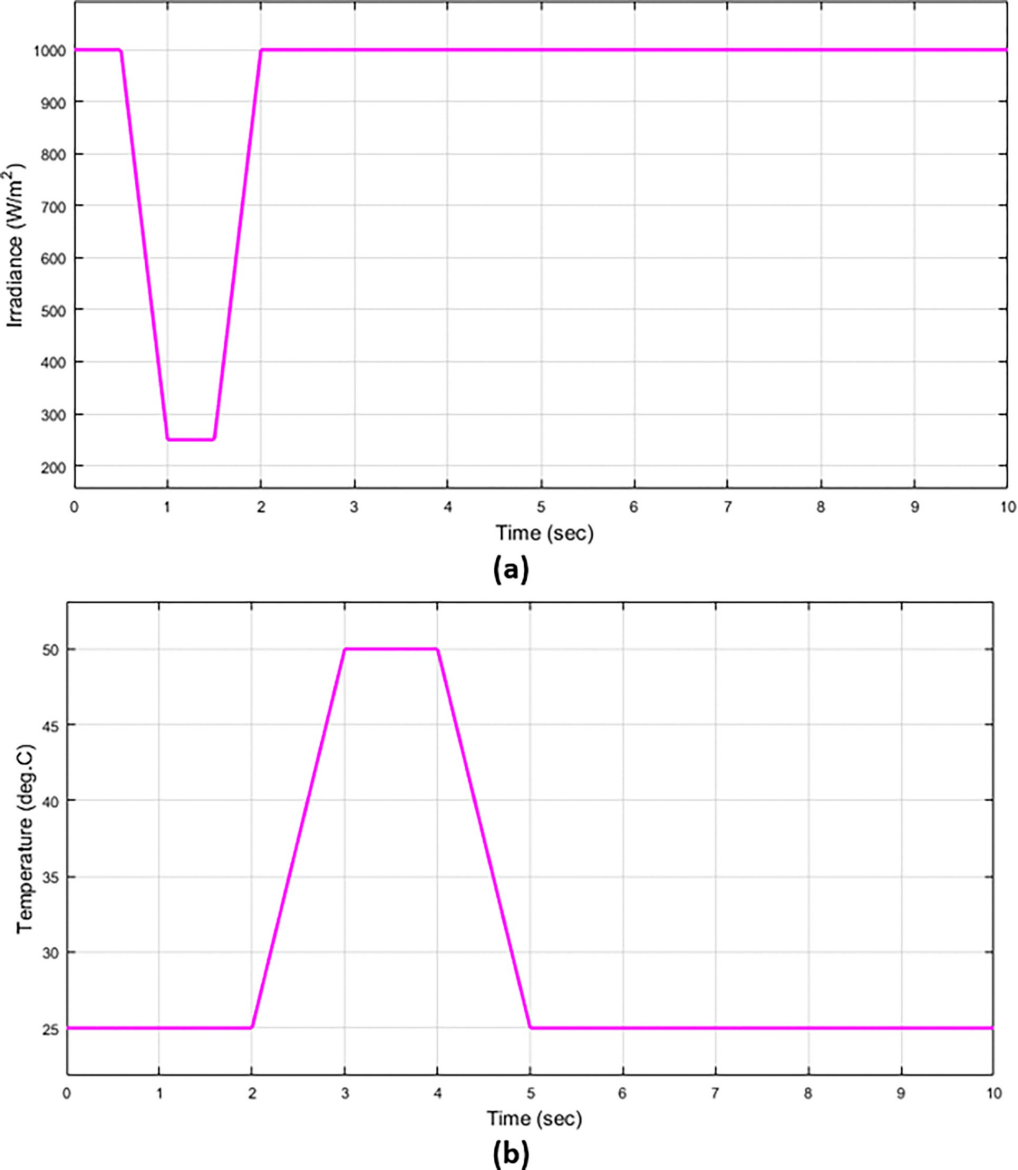
The irradiation and temperature in the PV system (a) Solar irradiance (b) temperature.

The appearance of the term “Hosting Capacity” coincided with the development of many control methods that were integrated into the electrical systems to achieve the highest percentage of renewable energy stations’ participation in feeding the grid while maintaining its stability. In this article, the system will be validated based on the Volt-VAr & Power Factor (PF) controls under different cases (with and without) faults. In addition, the proposed control algorithm will be validated with reality system parameters. The irradiation and temperature curves of the PV system are described in Fig 5. The irradiation and temperature change from 0 times to 5 sec then the temperature and irradiance are fixed at a certain value depending the on deterministic method.

### 5.1. Effect of irradiation and temperature

**Fig 6:**
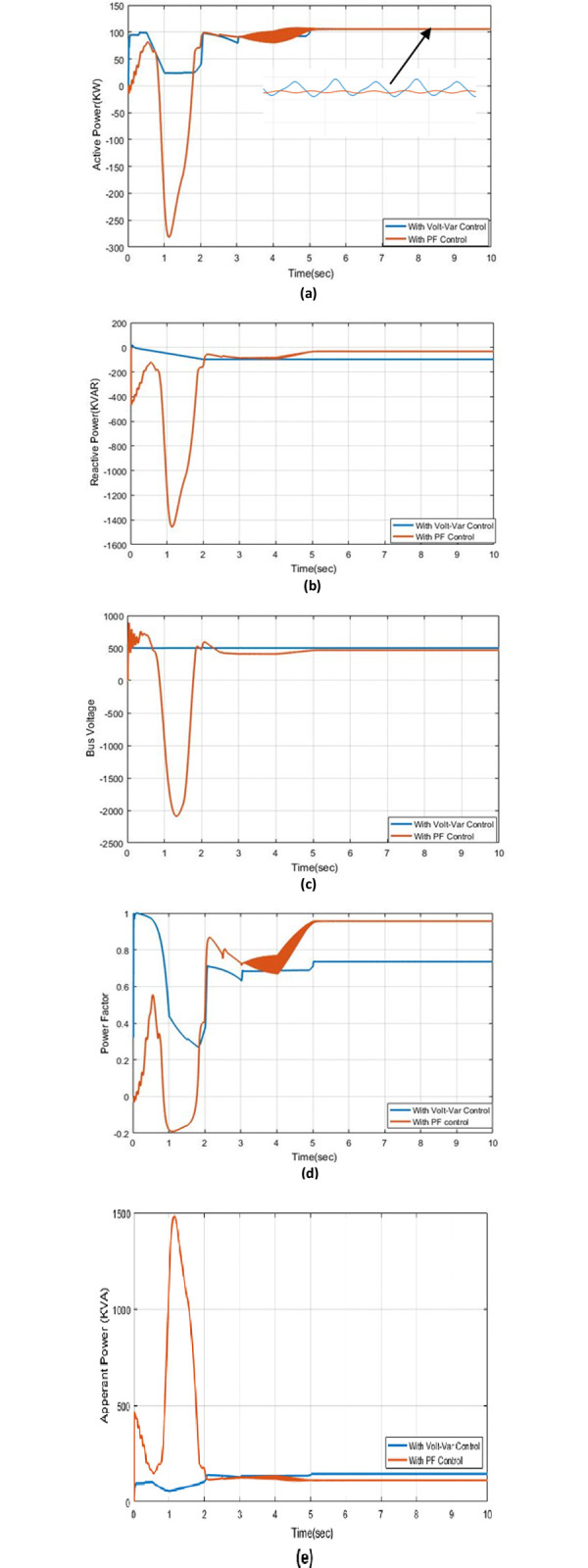
The outcome responses with Volt-Var and PF control strategies under effect of irradiation and temperature conditions (a) Real power (b) Reactive power (c) Bus voltage (d) Power factor (e) Apparent power.

The LV distribution network that supplies power to a photovoltaic system, with a capacity of 100 kVA and a combined load of 10 kVA located 5 km away from a feeder, has been modeled using MATLAB Simulink. The algorithm for power factor (PF) regulation is implemented in the system and compared to classical control to validate the effectiveness of the proposed approach. The results, including active power, reactive power, apparent power, bus voltage, and PF, with both Volt-VAr control and PF control, are presented in this study. In Fig 6A, it is observed that the active power remains constant with both Volt-VAr regulator and PF control under steady-state conditions. However, active power experiences changes during transient conditions due to variations in irradiance and temperature. Notably, the disturbance in PF control is more pronounced than in Volt-VAr control during transient conditions. Despite this, PF control exhibits small oscillations that stabilize over time, with a slight ripple. In contrast, the ripple in Volt-VAr control is larger during steady-state periods. Fig 6B illustrates reactive power with Volt-VAr control and PF control. It is noteworthy that the reactive power changes from (-97.3) kVAr in Volt-VAr control to (-32.4) kVAr in PF control. This reduction in reactive power absorption indicates an improvement in system stability and a decrease in voltage issues at the bus. The bus voltage is reduced to (466.5) V at steady-state to maintain system stability, as shown in Fig 6C. At steady-state conditions in Fig 6D, the power factor reaches a high value of (0.96) with PF control, whereas Volt-VAr control records a power factor of (0.74). The power factor is not constant during transient conditions, as real and reactive energy change due to variations in irradiance and temperature. Fig 6D also depicts apparent power changes, where Volt-VAr control absorbs more reactive power to adhere to the voltage bus limit, as described in Fig 6E.

### 5.2. Effect of single-line to ground fault

**Fig 7:**
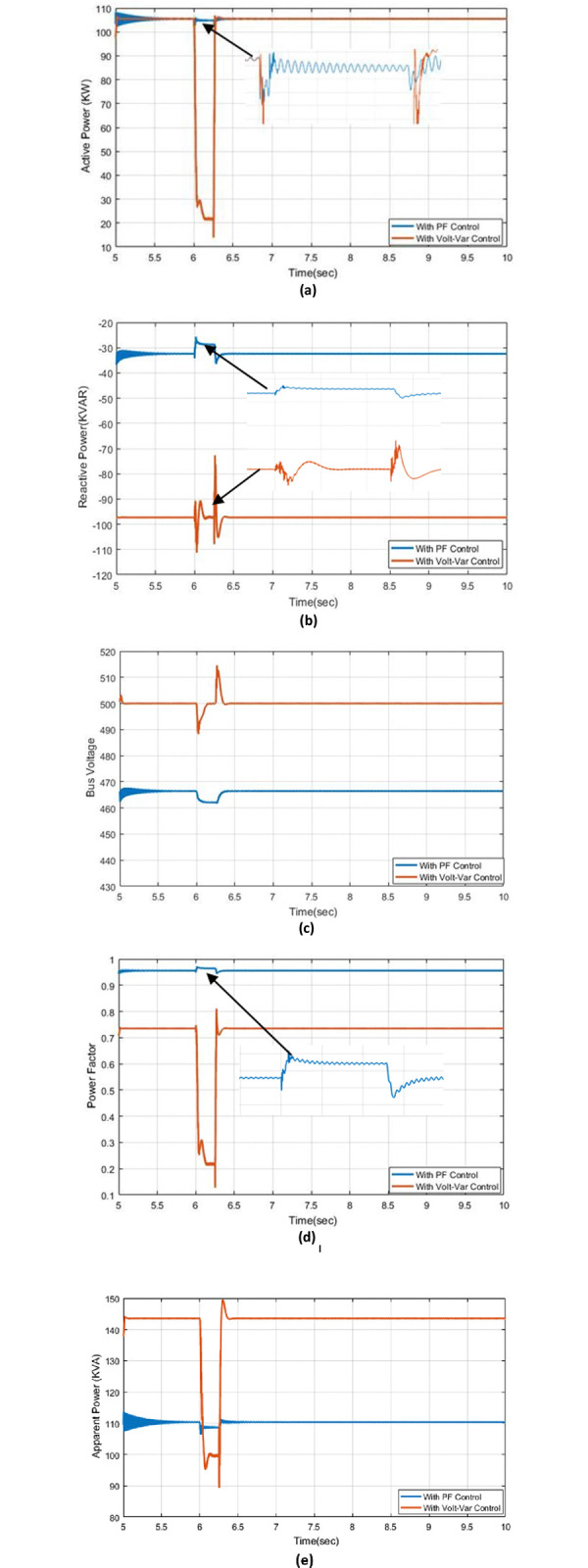
The outcome responses with Volt-Var and PF control strategies under effect of single-line to ground fault condition (a) Real power (b) Reactive power (c) Bus voltage (d) Power factor (e) Apparent power.

The results of actual power, reactive power, apparent power, voltage bus, and PF with two controls Volt-VAr regulator and PF control are described in Fig 7. The active power with PF control and Volt-VAr control at a single-to-ground fault at a time of 6 seconds is shown in Fig 7A. The fact that the disturbance in Volt-VAr control is greater than that in PF control indicates that the PF control has very little effect on the active power when a single line to ground fault occurs. Fig 7B depicts Volt-VAr regulation of reactive power and PF control. The disturbance in PF is also very small at fault when compared to Volt-VAr control. The fault affects the voltage bus in a connected PV system, but it has a smaller effect in PF control than in Volt-VAr control as demonstrated in Fig 7C. The behaviour response for PF is shown in Fig 7D and It is clear that the system is exposed to a severe disturbance when the fault occurs in the Volt-VAr control, as it has a severe drop of up to *0*.*2*. However, the response of PF control has a good performance with small disturbances. The apparent power with Volt-VAr control and PF control at a single fault to the ground are shown in Fig 7E, it’s also the disturbance with PF control is better than with Volt-VAr control, and finally, the results of actual power, reactive power, apparent power, bus voltage, and PF are good at PF control at occurring single-line to ground comparing with Volt-VAr control.

### 5.3. Effect of two-line to ground fault

**Fig 8:**
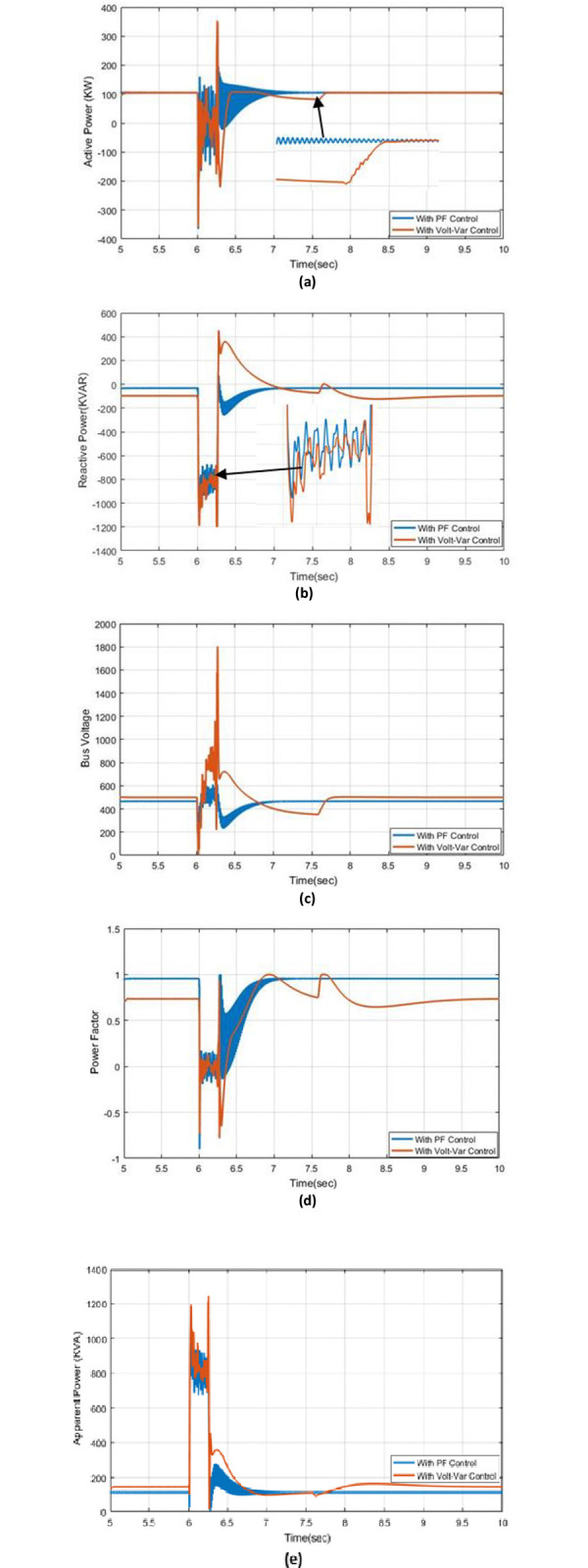
The outcome responses with Volt-Var and PF control strategies under effect of two-line to ground fault condition (a) Real power (b) Reactive power (c) Bus voltage (d) Power factor (e) Apparent power.

The results of real, reactive, apparent power, voltage bus, and PF with Volt-VAr control and PF control are displayed within this case. Fig 8A shows the active power with PF control and Volt-VAr control at a two-line-to-ground fault at a time of 6 seconds. It’s obvious that the disturbance on active power by using PF control is smoother than when using the Volt-VAr control and fast response to reach the steady-state condition, Fig 8B shows the reactive power under PF and Volt-VAr regulation. A comparison between the PF disturbance to Volt-VAr control, the fault is also very minor. The reactive power at the Volt-VAr condition has a long time to reach the steady-state condition comparing the PF control. it has a fast response to reach its steady-state position. The fault affects the voltage bus in a connected PV system, but it has a smaller effect in PF control than in Volt-VAr control as demonstrated in Fig 8C. The PF has different behavior under Volt-VAr and PF control, where the disturbance during fault is better at PF control than Volt-VAr control and the fast response to get to a steady-state position as described in Fig 8D, the apparent power with Volt-VAr control and PF control at s two-line fault to the ground are shown in Fig 8E, it’s also the disturbance with PF control is better than with Volt-VAr control, finally, the results active, reactive, apparent power, bus voltage, and PF are good at PF control at occurring two-line to ground comparing with Volt-VAr control.

### 5.4. Effect of three-line to ground fault

**Fig 9:**
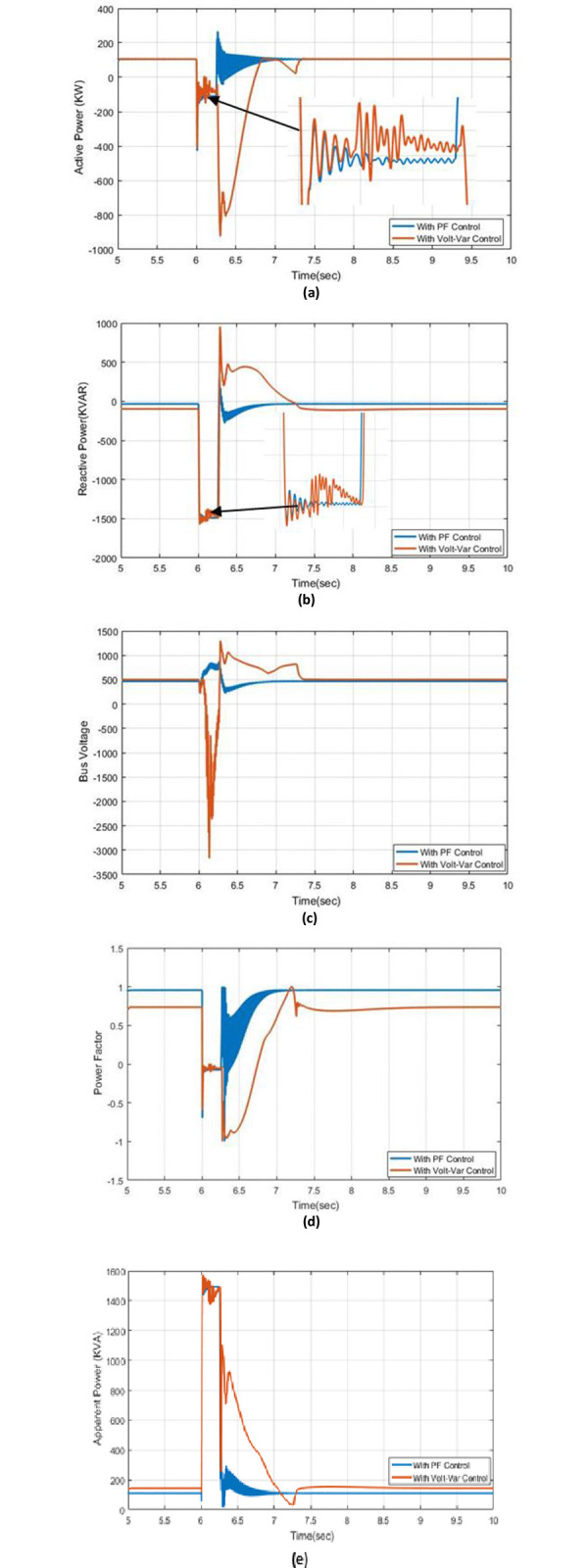
The outcome responses with Volt-Var and PF control strategies under effect of three-line to ground fault condition (a) Real power (b) Reactive power (c) Bus voltage (d) Power factor (e) Apparent power.

The results of three types of power: actual, reactive, and apparent, voltage bus, and PF with Volt-VAr control and PF control are demonstrated in this instance Fig 9A demonstrates the active power with PF control and Volt-VAr control at a three-line-to-ground fault at 6 seconds.

It’s obvious that the disturbance on active power by using PF control is smoother than when using the Volt-VAr control and fast response to reach the steady-state condition, Fig 9B shows the reactive power under PF and Volt-VAr control. A comparison between the PF disturbance to Volt-VAr control, the fault is also very minor. The reactive power at the Volt-VAr condition has a long time to reach the steady-state condition comparing the PF control it has a fast response to achieve a steady-state. The fault affects the voltage bus in a connected PV system, but it has a smaller effect in PF control than in Volt-VAr control as demonstrated in Fig 9C. The power factor has a good performance under control and the disturbance causing the fault is better at PF control than Volt-VAr control. The fast response to get to a steady-state situation under PF and Volt-VAr control is shown in Fig 9D. The apparent power with Volt-VAr control and PF control at three-line fault to the ground are shown in Fig 9E, it shows that the disturbance with PF control is better than Volt-VAr control, finally, the results real, reactive, apparent power, bus voltage, and PF are good at PF control at occurring three-line to ground comparing with Volt-VAr control.

## 6. Conclusion

The challenge faced by PV systems aiming to connect to the grid at low or medium voltage lies in the implementation of Volt-VAr and Power Factor (PF) controls, which are employed to enhance hosting capacity. This paper delves into various scenarios of PV system integration through the utilization of diverse inverter controls, specifically PF and Volt-VAr controls. In Case 1, a comparison is drawn between PF controls and Volt-VAr control in typical operational circumstances. Here, the application of PF and Volt-VAr controls leads to an augmented Hosting Capacity (HC), with the PF increasing from 0.74 under Volt-VAr control to 0.96 under PF control.

In Case 2, the focus shifts to a comparison between Volt-VAr and PF controls in the event of a lone line-to-ground fault at the connection bus. Notably, the disturbance impact introduced by Volt-VAr control is considerably greater than that observed with PF control. This indicates that the performance of the system is notably superior under PF control compared to Volt-VAr control.

Case 3 and Case 4 involve a comparison of different controls under scenarios of two lines-to-ground fault and three lines-to-ground faults, respectively. It becomes evident that PF control consistently outperforms Volt-VAr control in the event of any fault occurrence.

Future endeavors could involve expanding the study to encompass additional loads, similar loads with distinct consumer objectives, and the potential influence of dispersed generation. As the system grows larger and more dependent on infrastructure capacity and developmental conditions, future infrastructure plans might incorporate these methodologies to determine increased loading, distribution expansion, and the integration of dispersed generation. Such developments could necessitate more precise estimation techniques to address the intricate challenges associated with calculating hosting capacity.
